# P-315. Safety and Effectiveness of Nasal Povidone-Iodine Decolonization Among Patients on Hemodialysis: A Multicenter Stepped-Wedge Cluster Randomized Trial

**DOI:** 10.1093/ofid/ofae631.518

**Published:** 2025-01-29

**Authors:** Marin Schweizer, A M Racila, Anitha Vijayan, David A Pegues, Susan C Bleasdale, Jesse T Jacob, Amy O’Shea, Jason Cobb, Mony Fraer, Melissa Ward, Pam C Tolomeo, Joseph Kellogg, Brenna Lindsey, Fiona Armstrong-Pavlik, Kimberly C Dukes, Stacey Hockett-Sherlock, Alexandre Marra, Linda Boyken, Daniel Diekema, Loreen Herwaldt

**Affiliations:** School of Medicine and Public Health, University of Wisconsin-Madison, Madison, Wisconsin; Iowa City VA Center for Access and Delivery Research and Evaluation, Iowa City, Iowa; Intermountain Health, Salt Lake City, Utah; Hospital of the University of Pennsylvania, Philadelphia, Pennsylvania; University of Illinois at Chicago, Chicago, IL; Emory University School of Medicine, Atlanta, GA; University of Iowa Carver College of Medicine, Iowa City, Iowa; Emory University, Atlanta, Georgia; University of Iowa, Iowa City, Iowa; University of Iowa, Iowa City, Iowa; University of Pennsylvania, Philadelphia, Pennsylvania; Emory University, Atlanta, Georgia; University of Illinois at Chicago, Chicago, IL; University of Iowa, Iowa City, Iowa; University of Iowa Carver College of Medicine, Iowa City, Iowa; University of Iowa, Iowa City, Iowa; University of Iowa Hospital and Clinics, iowa city, Iowa; University of Iowa, Iowa City, Iowa; University of Iowa Hospitals and Clinics, Iowa City, Iowa; University of Iowa, Iowa City, Iowa

## Abstract

**Background:**

The *SHEA/IDSA/APIC Strategies to Prevent MRSA Transmission and Infection Practice Recommendations* advised that facilities consider decolonizing patients on hemodialysis. We implemented a nasal decolonization intervention in which patients self-administered povidone-iodine (PVI) at each dialysis session. We aimed to assess intervention safety and effectiveness.

**Figure:**
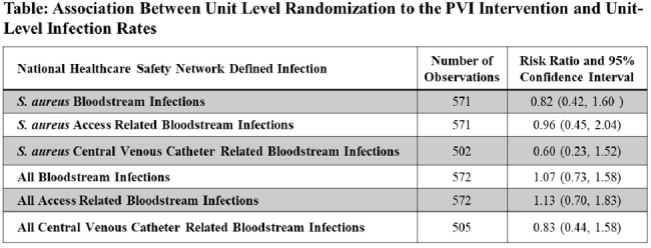

**Methods:**

We performed a stepped wedge cluster randomized trial at 16 outpatient hemodialysis units affiliated with 5 academic medical centers between 2020-2023. Adverse events were self-reported at 1 and 6 months. While the analysis was at the hemodialysis unit level, patients were required to give verbal informed consent for PVI use. Outcomes included National Healthcare Safety Network reportable dialysis events aggregated at hemodialysis unit level, including bloodstream infections (BSI), access-related BSI, and central venous catheter (CVC) BSI for all pathogens and for *Staphylococcus aureus* (SA). The primary outcome was SA BSI. A generalized linear mixed model with a negative binomial distribution, log link function, and an offset for person-months with a random intercept for each hemodialysis unit was performed.

**Results:**

Overall, 1,351 patients received hemodialysis at these centers each month. Of those, 362 patients verbally consented to use PVI. Among these, 3.9% reported side effects: nasal drip, congestion or burning/stinging, unpleasant smell, headache, or minor nose bleed. A reported side effect ‘yellow tears’ was assessed via chart review and resolved by discontinuing PVI. There were no statistically significant associations between unit level randomization to the PVI intervention and infections. However, there was a non-statistically significant trend toward a protective association between unit-level randomization to PVI and SA infections, particularly SA CVC BSI.(Table)

**Conclusion:**

Long-term nasal decolonization with PVI was safe with few adverse events. Unit level randomization to the PVI intervention did not significantly decrease unit-level infections. Given low patient enrollment and added infection prevention interventions due to COVID-19, the study could not determine if PVI decolonization could decrease BSI rates in the hemodialysis setting.

**Disclosures:**

**Marin Schweizer, PhD**, 3M: Grant/Research Support **Anitha Vijayan, MD**, Baxter: Honoraria|NxStage: Advisor/Consultant|Qanta: Honoraria **David A. Pegues, MD**, DaVita/Total Renal Care: Advisor/Consultant **Daniel Diekema, MD**, Affinity Biosensors: Grant/Research Support|bioMerieux, Inc: Grant/Research Support **Loreen Herwaldt, MD**, 3M: Grant/Research Support|PDI: Grant/Research Support

